# Pharmacogenomic association study on the role of drug metabolizing, drug transporters and drug target gene polymorphisms in drug-resistant epilepsy in a north Indian population

**DOI:** 10.4103/0971-6866.80357

**Published:** 2011-05

**Authors:** Ritu Kumari, Ram Lakhan, R. K Garg, J Kalita, U. K Misra, Balraj Mittal

**Affiliations:** Department of Genetics, Sanjay Gandhi Postgraduate Institute of Medical Sciences, Lucknow, Uttar Pradesh, India; 1 Department of Neurology, Sanjay Gandhi Postgraduate Institute of Medical Sciences, Lucknow, Uttar Pradesh, India; 2 Department of Neurology, CSM Medical University, Lucknow, Uttar Pradesh, India

**Keywords:** Drug resistance, epilepsy, pharmacogenomics

## Abstract

**BACKGROUND:**

In epilepsy, in spite of the best possible medications and treatment protocols, approximately one-third of the patients do not respond adequately to anti-epileptic drugs. Such interindividual variations in drug response are believed to result from genetic variations in candidate genes belonging to multiple pathways.

**MATERIALS AND METHODS:**

In the present pharmacogenetic analysis, a total of 402 epilepsy patients were enrolled. Of them, 128 were diagnosed as multiple drug-resistant epilepsy and 274 patients were diagnosed as having drug-responsive epilepsy. We selected a total of 10 candidate gene polymorphisms belonging to three major classes, namely drug transporters, drug metabolizers and drug targets. These genetic polymorphism included CYP2C9 c.430C>T (*2 variant), CYP2C9 c.1075 A>C (*3 variant), *ABCB1* c.3435C>T, *ABCB1*c.1236C>T, *ABCB1*c.2677G>T/A, *SCN1A* c.3184 A> G, *SCN2A* c.56G>A (p.R19K), GABRA1c.IVS11 + 15 A>G and GABRG2 c.588C>T. Genotyping was performed using polymerase chain reaction-restriction fragment length polymorphism (PCR-RFLP) methods, and each genotype was confirmed via direct DNA sequencing. The relationship between various genetic polymorphisms and responsiveness was examined using binary logistic regression by SPSS statistical analysis software.

**RESULTS:**

CYP2C9 c.1075 A>C polymorphism showed a marginal significant difference between drug resistance and drug-responsive patients for the AC genotype (Odds ratio [OR] = 0.57, 95% confidence interval [CI] = 0.32–1.00; *P* = 0.05). In drug transporter, *ABCB1*c.2677G>T/A polymorphism, allele A was associated with drug-resistant phenotype in epilepsy patients (*P* = 0.03, OR = 0.31, 95% CI = 0.10-0.93). Similarly, the variant allele frequency of *SCN2A* c.56 G>A single nucleotide polymorphism was significantly higher in drug-resistant patients (*P* = 0.03; OR = 1.62, 95% CI = 1.03, 2.56). We also observed a significant difference at the genotype as well as allele frequencies of GABRA1c.IVS11 + 15 A > G polymorphism in drug-resistant patients for homozygous GG genotype (*P* = 0.03, OR = 1.84, 95% CI = 1.05–3.23) and G allele (*P* = 0.02, OR = 1.43, 95% CI = 1.05–1.95).

**CONCLUSIONS:**

Our results showed that pharmacogenetic variants have important roles in epilepsy at different levels. It may be noted that multi-factorial diseases like epilepsy are also regulated by various other factors that may also be considered in the future.

## Introduction

Epilepsy is a common, serious, but treatable neurological disorder, affecting at least 60 million people worldwide.[1] Several pharmacological agents are available in the market for the treatment of epilepsy. These anti-epileptic drugs (AEDs) increase inhibition, decrease excitation or prevent aberrant burst-firing of the neurons.[1] However, 20–30% of the epilepsy patients do not respond adequately to the currently available AEDs, and there is high incidence of adverse drug reactions.[2] Therefore, drug resistance and adverse reactions are important clinical problems in the treatment of epilepsy. Large interindividual variation in efficacy and adverse effects of anti-epileptic therapy presents opportunities and challenges in newly emerging areas of pharmacogenomics. In the post genomic era, the interindividual variations in drug response have been attributed to allelic variations in the genes.[3–5] These genetic variations can potentially affect the individual responsiveness to the drug at several steps, which include drug absorption, drug distribution, drug metabolism, drug elimination and drug concentration at target sites.[6] There is a growing list of polymorphisms found in different classes of genes encoding drug-metabolizing enzymes (DMEs), drug transporters, receptors and drug targets, which have been linked to drug effects in humans. In pharmacotherapy, drug metabolism represents a prominent pathway both in qualitative and quantitative elimination of drugs, including AEDs. It is accomplished by the hepatic (metabolism) and/or renal (excretion) route, which comprises the so-called phase I [(e.g., oxidative reactions catalyzed by various cytochrome P-450 enzymes (CYPs)] and phase II (e.g., conjugations like glucuronidation) reactions. The CYP family comprises major phase-I DMEs, and is responsible for the metabolism of many commonly prescribed AEDs. Variants with very high enzymatic activity may be associated with a need for higher drug dosages than usually prescribed, but low or absence of biotransformation capacity may result in treatment failure due to inadequate drug levels.[7] Thus, there are a variety of potential mechanisms by which polymorphic drug metabolism can affect AED responsiveness.[8] Several classes of drug transport proteins are also shown to have a pharmacogenetic relevance. The ABC proteins are products of multidrug resistance (MDR1/*ABCB1*) genes that are expressed widely, including capillary endothelial cells that constitute the blood–brain barrier responsible for ATP-mediated efflux of different compounds outside the brain, serving as a defense mechanism.[9,10] The *ABCB1* (MDR1) gene codes for prototype molecule P-glycoprotein (Pgp) that is recognized to be a key element in regulating access of a diverse array of therapeutic agents to the brain and other tissues.[11,12] Several commonly used AEDs have been proposed to be substrates for P-gp-mediated transport.[13] A large number of genetic variations have been documented in human *ABCB1*, and some of them have been shown to the influence dosage of AEDs. The role of voltage-gated and ligand-gated ion channels in epileptogenesis of both genetic and acquired epilepsies, and as targets in the development of new AEDs, is very important.[14] Ionic currents generated through sodium channels are inhibited by a number of different types of therapeutically important AEDs. Several single nucleotide polymorphisms (SNPs) in the sodium channel genes have been described so far, but only a few, including *SCN1A* p. Thr1067Ala or c.3184 A>G (rs2298771) and *SCN2A* p.Arg19Lys or c.56 G>A (rs17183814) gene polymorphisms have been found to have a functional significance in the different neurological disorders.[15] In the central nervous system, γ-Aminobutyric acid (GABA) is the major inhibitory neurotransmitter that controls neuronal excitability and network interactions in the cerebral cortex of the brain. AEDs such as benzodiazepines, phenobarbital, gabapentin and topiramate are important targets of the GABAA receptor.[16] Recently, it has been reported that AED-resistant rats differ from drug-responsive rats in GABAA receptor subunit expression in a rat model of temporal lobe epilepsy. Therefore, genetic variations in *GABAA* receptor subunits may also be involved in resistance to AEDs.[17] In this way, individual genotype influences almost all stages of pharmacokinetics (hepatic drug-metabolizing enzymes, membrane drug transporters) and pharmacodynamics (sodium channels, GABA receptors, etc.).[18] Therefore, the present study was planned to study the genetic polymorphisms in genes for cytochrome P450, multidrug transporter, voltage-gated sodium ion channels and ligand-gated GABAA receptor genes in drug responsiveness in patients undergoing treatment for epilepsy.

## Materials and Methods

The study was retrospective and consisted of patients with epilepsy from northern a Indian population. Patients were recruited from the outpatient department (OPD) of Neurology attending the clinics of SGPGIMS and CSMMU Medical University in Lucknow. The patients were subjected to detailed clinical examination, investigations and clinical and family history to ascertain the type of epilepsy and treatment protocol based on clinical proforma. Classification of epilepsy was based on criteria instituted by the International League against Epilepsy (ILAE). For follow-up patients, some of the information was taken from their hospital records. The records of medication, dose, adverse reaction and seizure frequency was maintained for each patient. Any change in medication, its dose and number of seizures was followed. Blood sample was collected from nonresponders and responder patients fulfilling stringent criteria of drug responsiveness and adverse drug reactions. Serum drug levels were determined in 20% of the cases to ascertain drug compliance, especially in those showing signs of adverse drug reactions (ADRs) or nonresponsiveness. This study was approved by the local ethics committees of both the participating institutions. The clinical profile of the drug-responsive and the drug-resistant epilepsy patients was based on hospital investigations. Initial diagnosis was based on two or more unprovoked seizures in the individuals. Both new and old patients were taken after informed consent.

### 

#### Definition of drug resistance and responsiveness

The main criterion for drug resistance was the occurrence of at least four seizures over a period of 1 year, with three appropriate AEDs at maximum tolerated doses.[19,20] Patients who had undergone surgery for seizure control were considered refractory, irrespective of their outcome after surgery. The epilepsy patients who had complete freedom from seizures for at least 1 year from the last follow-up visit were considered drug responsive.

#### Sample DNA extraction

A venous blood sample (5 ml) was collected from each subject and was kept frozen till DNA extraction. Genomic DNA was isolated from peripheral blood leukocytes by the salting-out protocol.[21] Extracted DNA was quantified using a Nanodrop Analyzer (ND-1000) spectrophotometer (Nano Drop Technologies Inc., Wilmington, DE, USA). The plasma was separated and stored at -20°C for drug level assay.

#### Selection of candidate genes

The candidate genes were selected on the basis of their functional role, current biological knowledge of epilepsy, a reported prevalence of at least 5% for the variant allele and published evidence of an association with epilepsy and other neurological disorders. We aimed to select, as candidate genes, major drug targets (voltage and ligand gated), multidrug transporters (*ABCB1*) and metabolizers (CYP2C9*2 and CYP2C9*3) of the principal AEDs for use in future pharmacogenetic studies. Details of candidate genes selected for the study are given in Table 1.

**Table 1:** Candidate genes, their activity and polymorphic sites

Genes	Variant/ mutantallele	Site of polymorphism/effect	Change activity
CYP2C9	C (*2)	430C>T(Arg144Cys, Exon-3)	Decreased
CYP2C9	T (*3)	1075 A>C (Ile359 Leu, Exon-7)	Decreased
*ABCB1*	C	1236 C>T (Exon-12)	C allele associated with higher expression
*ABCB1*	T and A	2677G>T/A (Exon- 21)	Ala 893 Ser; no amino acid change
*ABCB1*	C	3435 C>T (Exon-26)	Low activity
SCN1A	G	3184 A>G (IVS11+15)	Altered membrane excitability
SCN2A	A	c.56 G>A (Exon-2)	Altered membrane excitability
GABRA1	G	IVS11+15 A>G (Intron-11)	Intronic
GABRG2	T	588 C>T (Exon-5)	Synonymous

#### Genotyping of genetic markers used in the study

The genotypes were determined by the PCR-RFLP method. Primers, annealing temperature, amplified fragment size, restriction pattern and restriction enzymes used are listed in Table 2.

**Table 2:** Primer sequences, amplicon size, restriction enzyme, annealing temperature and restriction pattern of various polymorphisms studied

Gene	Primer Sequence	Amplicon size	Annealing Temperature	Restriction enzyme and Restriction pattern (bp)	Reference
CYP2C9(*2) (430 C>T)	5’-CACTGGCTGAAAGAGCTAACAGAG-3’	372bp	620C for 45 sec	*Sau*96I/ 179, 119, 74 and 253bp	
	5’-GTGATATGGAGTAGGGTCACCCAC-3’				(Aynacioglu *et al*.,1999)[22]
CYP2C9(*3) (1075 A>C)	5’-AGGAAGAGATTGAACGTGTGA-3’	130bp	570C for 45 sec	*StyI*/130,104 and 26 bp	
	5’- GCAGGCTGGTGGGGAGAAGGCCAA -3’				
ABCB1 3435C>T	5’ACTCTTGTTTTCAGCTGCTTG-3’	231bp	560C for 20sec	*DpnII*/163,68 and 231bp	(Hamdy et.al, 2003)[23]
	5’ AGAGACTTACATTAGGCAGTGACTC-3’				
ABCB1 1236C>T	5’-TATCCTGTGTCTGTGAATTGCC-3’	315bp	630C for 1 min	*BsuRI*/205, 140, 35,240,and 35 bp	(Cascorbi *et al*., 2001)[24]
	5’-CCTGACTCACCACACCAATG-3’				
ABCB1 2677G>T	5,TGCAGGCTATAGGTTCCAGG-3’	224bp	600C for 30 sec	*BanI*/224,198,and 26bp	(Cascorbi *et al*., 2001)[24]
	5’-TTTAGTTTGACTCACCTTCCCG-3’				
ABCB1 2677G>A	5,TGCAGGCTATAGGTTCCAGG-3’	220bp	600C for 30 sec	*RsaI*/220,206 and 14 bp	(Cascorbi *et al*., 2001)[24]
	5’- GTTTGACTCACCTTCCCAG -3’				
SCN1A 3184 A>G	5’-TGCACAAAGGAGTAGCTTATG-3’	168bp	570C for 30 sec	*PvuII*/168,145,and 23bp	(Chou IC, *et al*. 2003)[25]
	5’-AGTCAAGATCTTTCCCAATTTCAG-3’				
SCN2A 56 G>A	5’-AATCACCTTTTATTCTAATGGTC-3’	206bp	600C for 30 sec	*ScrFI*/178,130, 64. 206and 28bp	(Haug *et al*., 2001)[26]
	5’-CAGTGAAGGCAACTTGACTAAGA-3’				
GABRA1 IVS11 + 15 A > G	5’-GCT ATG GAT TGG TTT ATT GCC GTG TG3’	165bp	600C for 30 sec	*AvaII*/165,and141bp	Park CS *et al*., 2006[27]
	5’- ATA ATA TTG ATG TAC TAC AGG GAC-3’				
GABRG2 588C > T	5’-AATCACCTTTTATTCTAATGGTC-3’	122bp	570C for 45 sec	*ApoI*/122,102 and 20bp	Chou IC *et al*., 2007[28]

#### Statistical analysis

The sample size was calculated using the QUANTO 1.1 program (hydra.usc.edu/gxe). The desired power of our study was set at 80%. Relative risks for power calculation were set at 2. Descriptive statistics of patients and controls were presented as the mean and standard deviations (SDs) for continuous measures, while frequencies and percentages were used for categorical measures. The relationship between various genotypes and responsiveness was examined using the binary logistic regression. Association was expressed as odds ratios (OR) or risk estimates with 95% confidence intervals (CI). The association was considered to be significant when the *P*-value was <0.05.

## Results

A total of 402 epilepsy patients were enrolled in the study. Based on inclusion and exclusion criteria, 128 were diagnosed as multiple drug-resistant epilepsy and 274 patients had drug-responsive epilepsy [Table 3]. Among the patients, 70.8% were male and 29.2% were female. The mean age ± SD year of the patients was 24.30 ± 11.6 years, with no significant differences between the drug-resistant (23.8 ± 11.7) versus the drug-responsive (24.5 ± 11.5) patients. The mean age at onset of the first seizure was 17.04 ± 10.9 years for the responders and 14.60 ± 10.4 years for the nonresponders, and differed significantly between the patient groups (*P* = 0.04). AED levels were also measured in 100 patients to confirm drug compliance. The mean carbamazepine, phenytoin and valproate levels were 8.26 ± 5.25, 11.27 ± 8.12 and 68.0 ± 36.22 µg/ml, respectively.

**Table 3:** Demographic profiles of epilepsy patients

Sex	Responder (274)	Nonresponder (128)
Male	194 (70.8%)	95 (74.2%)
Female	80 (29.2%)	33 (25.8%)
Age (years) ± SD	24.5 ± 11.5	23.8 ± 11.7
Age of onset	17.04 ± 10.9	14.60 ± 10.4

### 

#### CYP2C9*2 and CYP2C9*3 polymorphisms and drug resistance

In our study population, none of the individuals were homozygous for 2C9*2 (430 C>T) or 2C9*3(1075 A>C) [Table 4]. No significant differences were observed at the allele level for the 2C9*2 430 T variant (OR = 0.67, 95% CI = 0.28–0.1.50, *P* = 0.36) [Table 4]. *CYP2C9* 1075 A>C (*3 variant) polymorphism showed a marginally significant difference between patients having multiple drug resistance and drug-responsive patients for AC genotype (OR = 0.57, 95% CI = 0.32–1.00, *P* = 0.05). The CYP2C9*3 variant allele frequency was also a little higher in drug-responsive patients compared with drug-resistant patients (*P* = 0.06, OR = 0.60, 95% CI = 0.35–1.03), and seems to be contributing toward a lower risk for developing multiple drug resistance in epilepsy [Table 4].

**Table 4:** Distribution of CYP2C9*2 (430 C>T) and CYP2C9*3 (1075 A>C) gene polymorphism in drug-resistant and drug-responsive patients with epilepsy

Genotype	Drug-resistant N = 128 (%)	Drug-responsive N = 274 (%)	OR (95% CI)	*P*-value
CYP2C9*2 (430 C>T)				
430 CC	121 (94.5)	252 (92.0)	Reference	-
430 CT	7 (5.5)	22 (8.0)	0.66 (0.27–1.59)	0.35
430 TT	-	-	-	-
430 C*	249 (97.3)	526 (96.0)	Reference	-
430 T*	7 (2.7)	22 (4.0)	0.67 (0.28–1.50)	0.37
CYP2C9*3 (1075 A>C)				
1075 AA	109 (85.2)	210 (76.6)	Reference	-
1075 AC	19 (14.8)	64 (23.4)	0.57 (0.32–1.00)	0.05
1075 CC	-	-	-	-
1075 A*	237 (92.6)	484 (88.3)	Reference	-
1075 C*	19 (7.4)	64 (11.7)	0.60 (0.35–1.03)	0.06

* denotes alleles. Significant *P*-values are shown in bold

#### *ABCB1* polymorphisms and drug resistance

The genotype frequencies of *ABCB1* 3435C>T did not differ significantly in the drug-resistant versus the drug-responsive patients for CT (*P* = 0.06, OR = 1.95, 95% CI = 0.96–3.96) and TT (*P* = 0.18, OR = 1.64, 95% CI = 0.79–3.41) genotypes [Table 5]. No significant differences were observed at the allele level also (*P* = 0.39, OR = 1.14, 95% CI = 0.84–1.54). In the *ABCB1 c.2* 677G>A polymorphism, allele “A” was associated with the drug-resistant phenotype in epilepsy patients (*P* = 0.03, OR = 0.31, 95% CI = 0.10–0.93). However, genotype frequencies of 2677G>T *ABCB1* polymorphism did not differ significantly in the drug-resistant versus the drug-responsive epilepsy patients for GT genotype (*P* = 0.71, OR = 1.14, 95% CI = 0.55–2.36), for TT genotype (*P* = 0.87, OR = 1.06, 95% CI = 0.49–2.26) and for AT genotype (*P* = 0.39, OR = 0.53, 95% CI = 0.13–2.24) [Table 5]. Similarly, genotype and allele frequencies of 1236C>T *ABCB1* polymorphism also did not differ significantly in the drug-resistant and drug-responsive groups for the CT (*P* = 0.32, OR = 1.40, 95% CI = 0.62–2.61) and TT (*P* = 0.50, OR = 1.27, 95% CI = 0.77–1.42) genotypes [Table 5].

**Table 5:** Distribution of *ABCB1* 2677G>T/A (rs2032582), 1236C>T (rs1128503) and 3435C>T (rs1045642) gene polymorphism in drug-resistant and drug-responsive patients with epilepsy

Genotype	Drug-resistant N = 125 (%)	Drug-responsive N = 260 (%)	OR (95% CI)	*P*-value
2677G>T/A(rs2032582)				
GG	13(10.4)	28(10.8)	Reference	-
GT	47(52.8)	124(47.7)	1.14(0.55–2.36)	0.71
TT	41(32.8)	83(31.9)	1.06(0.49–2.26)	0.87
GA	2(1.6)	13(5.0)	0.33(0.06–1.68)	0.18
AT	3(2.4)	12(4.6)	0.53(0.12–2.24)	0.39
AA	None	-	-	-
G*	94(37.6)	193(37.1)	Reference	
T*	119(60.8)	301(57.9)	1.03(0.75–1.42)	0.82
A*	4(1.6)	26(5.0)	0.31(0.10–0.93)	0.03
1236C>T(rs1128503)				
CC	14(11.2)	38(14.6)	Reference	-
CT	70(56.0)	135(51.9)	1.40(0.71–2.77)	0.32
TT	41(32.8)	87(33.5)	1.27(0.62–2.61)	0.50
C*	98(39.2)	211(40.6)	Reference	
T*	152(60.8)	309(59.4)	1.05(0.77–1.42)	0.71
3435C>T(rs1045642)				
CC	12(9.6)	42(16.2)	Reference	-
CT	67(53.6)	120(46.2)	1.95(0.96–3.96)	0.06
TT	46(36.8)	98(37.7)	1.64(0.79–3.41)	0.18
C*	137(54.8)	302(58.1)	Reference	
T*	113(45.2)	218(41.9)	1.14(0.84–1.54)	0.39

* alleles

#### SCN2A (rs17183814) and SCN1A (rs2298771) polymorphism in drug-resistant epilepsy

The genotype or allelic frequencies of *SCN1A* c.A3184G polymorphism did not differ significantly in drug-resistant versus drug-responsive epilepsy patients for AG (*P* = 0.98, OR = 0.99, 95% CI = 0.62–1.58) or GG (*P* = 0.78, OR = 1.17, 95% CI = 036–3.78) [Table 6]. Similarly, genotype frequencies of *SCN2A* c.R19K also did not differ significantly in the drug-resistant versus the drug-responsive patients for RK (*P* = 0.97, OR = 1.44, 95% CI = 0.84–2.48) and KK (*P* = 0.09, OR = 3.49, 95% CI = 0.81–14.97) genotypes [Table 6]. However, the frequency of the K allele was significantly higher in the drug-resistant patients with epilepsy versus the drug-responsive patients (*P* = 0.03, OR = 1.62, 95% CI = 1.03–2.56). This suggests that *SCN2A* c.R19K polymorphism may be involved in modulating the drug response in epilepsy patients.

**Table 6:** Distribution of *SCN1A* c.3184 A>G (rs2298771) and *SCN2A* c.56 G>A (rs17183814) gene polymorphism in drug-resistant and drug-responsive patients with epilepsy

Genotype	Drug resistant N = 117 (%)	Drug responsive N = 219 (%)	OR (95% CI)	*P*-value
*SCN1A* c. 3184 A>G (rs2298771)				
AA	50 (42.7)	94 (42.9)	Reference	-
AG	62 (53.0)	117 (53.4)	0.99 (0.62–1.58)	0.98
GG	5 (4.3)	8 (3.7)	1.17 (0.36–3.78)	0.78
A*	162 (69.2)	305 (69.6)	Reference	-
G*	72 (30.8)	133 (30.4)	1.01 (0.72–1.43)	0.91
*SCN2A* c. 56G>A (rs17183814)				
RR	83 (70.9)	174 (75.9)	Reference	-
RK	29 (24.8)	42 (19.2)	1.44 (0.84–2.48)	0.97
KK	5 (4.3)	3 (1.4)	3.49 (0.81–14.97)	0.09
R*	195 (83.3)	390 (89.0)	Reference	-
K*	39 (16.7)	48 (11.0)	1.62 (1.03–2.56)	0.03

#### GABRA1 (rs2279020) and GABRG2 (rs211037) polymorphism in drug-resistant epilepsy

We observed a significant difference at the genotype as well as allele frequencies of *GABRA1 A>G* polymorphism in drug-resistant versus drug-responsive epilepsy patients for homozygous variant GG genotype (*P* = 0.03, OR = 1.84, 95% CI = 1.05–3.23) and G allele (*P* = 0.02, OR = 1.43, 95% CI = 1.05–1.95) [Table 7]. However, in *GABRG2*, 588C>T did not show any significant differences in drug-resistant versus drug-responsive epilepsy patients either at the genotype or the allele levels [Table 7]. This suggests that *GABRA1* IVS11 + 15 A>G intronic polymorphism may be involved in the drug response in epilepsy patients.

**Table 7:** Distribution of *GABRA1* (rs2279020) and *GABRG2* (rs211037) gene polymorphism in drug-resistant and drugresponsive patients with epilepsy

Genotype	Drug resistant N = 122 (%)	Drug responsive N = 259 (%)	OR (95% CI)	*P*-value
*GABRA1* c.IVS11 + 15 A>G (rs2279020)				
AA	34 (27.86)	92 (35.52)	Reference	Reference
AG	47 (38.52)	107 (41.31)	1.18 (0.70–2.00)	0.517
GG	41 (33.60)	60 (23.16)	1.84 (1.05–3.23)	0.031
A*	115 (47.13)	291 (56.18)	Reference	Reference
G*	129 (52.87)	227 (43.82)	1.43 (1.05–1.95)	0.020
*GABRG2* c.588C>T (rs211037)				
RR	66 (54.09)	137 (52.89)	Reference	Reference
RK	53 (43.44)	109 (42.08)	1.01 (0.65–1.57)	0.967
KK	3 (2.45)	13 (5.01)	0.48 (0.13–1.74)	0.263
R*	185 (75.81)	383 (73.94)	Reference	Reference
K*	59 (24.18)	135 (26.06)	0.91 (0.64–1.29)	0.578

## Discussion

Multiple genes are known to be responsible for drug responsiveness. For example, DMEs[29] include mainly cytochrome P450, drug transporters (MDR1) and drug targets, which include sodium channels, potassium channels and GABA receptors. In CYP450 genes related to the metabolism of AEDs, we observed the protective effects of mutant alleles of CYP2C9*2 (430 C>T) and CYP2C9*3 (1075 A>C) in epilepsy patients developing multiple drug resistance. These findings indicate the important role of *CYP2C9* variants in conferring a multiple drug-resistant phenotype against AEDs. Our findings are in support of previous observations that identified *CYP2C9* as a major drug metabolizer for commonly prescribed AEDs.[22] *CYP2C9* is responsible for the hydroxylation of up to 90% of serum phenytoin. Among AEDs, phenytoin, carbamazepine and phenobarbital mainly undergo significant metabolism by cytochrome P450 isozymes. Earlier studies have also observed a strong association between *CYP2C9* genotype and phenytoin maintenance dose requirement.[30–31] Weide *et al*.[30] found that *CYP2C9* allelic variants affect phenytoin dose requirement; for patients carrying at least one mutant *CYP2C9* allele, the mean phenytoin dose required to achieve a therapeutic serum concentration was about 37% lower than the mean dose required by wild-type individuals. Another similar study from Taiwan revealed that the *CYP2C9* and *CYP2C19* polymorphisms have dramatic effects on the population pharmacokinetic parameters of phenytoin.[32] On the basis of these observations, we propose that poor metabolizer phenotype could have an advantage over the extensive metabolizer phenotype in epilepsy pharmacotherapy. It is likely that patients with poor metabolizer phenotype will require lesser drug doses to control seizures as compared with epilepsy patients with extensive metabolism. Therefore, patients with extensive metabolism are more likely to become drug resistant during the course of antiepileptic treatment. It is now accepted that drug resistance is a complex phenotype, resulting from contribution of numerous genes. In addition to DMEs, differences in intestinal and blood–brain barrier multidrug transporters, *MDR1* (*ABCB1*) and *MRP2* expression also influence carbamazepine and phenytoin disposition and may account for inter-individual pharmacokinetic variability.[33] P-gp is involved in the transport of multiple drugs, and most AEDs are substrates for this protein. Over-expression of P-gp has been reported in endothelial cells isolated from the temporal lobe blood vessels of medically intractable epilepsy patients. Various genetic variations in the *ABCB1* gene have been linked to the difference in the drug responsiveness.

In this study, we analyzed three genetic polymorphisms of *ABCB1* 3435C > T, 1236C > T and 2677G > T/A polymorphisms but failed to find a significant association with these polymorphic variations in refractory epilepsy in the north Indian population. A number of studies from different populations have also explored the role of these polymorphisms in *ABCB1* genes on drug-refractory epilepsy, but have shown conflicting results. Many factors may be attributed for the nonuniformity and nonreplication of results from various studies, including low sample size and genetic heterogeneity within the groups. Also, there is no consensus on a single definition of intractability. Individual studies use different definitions, creating difficulties for comparisons of results across studies. We have used inclusion criteria for responders and nonresponders that were similar and consistent with the original study by Siddiqui *et al*.[19] Although all definitions are significantly associated with longer-term outcome, no single preferred definition of intractable epilepsy exists.[34] There needs to be consensus for a single definition of drug resistance in epilepsy so that results from different studies can be easily compared. Until now, very few studies have correlated drug targets like sodium channels in drug resistance and therapeutic dosage in patients with epilepsy. Therefore, we looked for an association of the two polymorphisms with drug-resistance phenotype in our patient groups. Even though *SCN1A* c.3184 A>G polymorphism was associated with generalized epilepsy,[35] it showed no influence on the multidrug resistance phenotype in patients with epilepsy. Similarly, Kwan *et al*.[36] also found no involvement of this polymorphism in drug-resistant epilepsy. However, *SCN1A* IVS5-91 G>A intronic polymorphism of the same gene has been reported to show a population-specific association with carbamazepine and multiple drug-resistance epilepsy.[37–39] It is now well established that various AEDs mediate their action through GABA binding.[40] It is also hypothesized that target receptor sites are somehow altered in the epileptic brain so that they are much less sensitive to the administered AEDs. In the present study, we found the involvement of *GABRA1* IVS11 + 15 A>G polymorphism in modulating drug response in pharmacotherapy, while *GABRG2* 588C>T was not found to be associated with drug resistance in north Indian epilepsy subjects. Several mutations in this gene have also been reported to be involved in epilepsy causation that result in loss of function of GABAA receptors via a reduction in GABA expression and accelerated deactivation.[41] It is possible that the association of *GABRA1* IVS11 + 15 A>G polymorphism with refractory phenotype in our study may occur due to changes in the structure and function of inhibitory GABAA receptors.[42] Excessive glutamate excitation and activation of drug resistance genes may also contribute to changes in the GABA receptor conformation and loss of drug efficacy. In the above-mentioned facts, we have provided evidence that genetic variations in all three classes of genes have some role in multiple drug resistance; but, it would be desirable to replicate them in larger cohorts. It should also be kept in mind that the overall genetic effect of these genes may have a greater role in determining the drug responsiveness rather than a single gene and its genetic polymorphisms alone. Response to newly administered AED treatments is highly dependent on past treatment history.[43] Other factors such as type of epilepsy, duration of seizure and no seizure prior to initiation of drug therapy[43] may also be responsible for differences in drug responsiveness. Therefore, the current studies associating particular genes and their variants with seizure control or adverse events have inherent weaknesses, yet it is believed that future pharmacogenomic studies with clearly defined phenotypes involving a multicenter approach will result in therapeutic application.
